# Toll-Like Receptor-4 Inhibitor TAK-242 Attenuates Motor Dysfunction and Spinal Cord Pathology in an Amyotrophic Lateral Sclerosis Mouse Model

**DOI:** 10.3390/ijms18081666

**Published:** 2017-08-01

**Authors:** Avi Fellner, Yael Barhum, Ariel Angel, Nisim Perets, Israel Steiner, Daniel Offen, Nirit Lev

**Affiliations:** 1 Department of Neurology, Rabin Medical Center, Beilinson Hospital, 39 Jabotinski St., Petah Tikva 49100, Israel; israels2@clalit.org.il (I.S.); niritle@clalit.org.il (N.L.); 2 Sackler Faculty of Medicine, Tel-Aviv University, Tel Aviv 69978, Israel; 3 Laboratory of Clinical Neuroscience, Felsenstein Medical Research Center, Rabin Medical Center, Petah Tikva 49100, Israel; YAELBA1@clalit.org.il; 4 Felsenstein Medical Research Center, Sackler Faculty of Medicine, Sagol School of Neuroscience, Tel-Aviv University, Tel-Aviv 69978, Israel; Arielangel1@post.tau.ac.il (A.A.); Nisimperets@mail.tau.ac.il (N.P.); doffen@post.tau.ac.il (D.O.)

**Keywords:** ALS, animal model, innate immune system, neuroinflammation, SOD1 mice, TAK-242, TLR4

## Abstract

Neuroinflammation contributes to amyotrophic lateral sclerosis (ALS) progression. TLR4, a transmembrane protein that plays a central role in activation of the innate immune system, has been shown to induce microglial activation in ALS models. TLR4 is up-regulated in the spinal cords of hSOD1^G93A^ mice. We aimed to examine the effects of specific TLR4 inhibition on disease progression and survival in the hSOD1^G93A^ mouse model of ALS. Immunologic effect of TLR4 inhibition in vitro was measured by the effect of TAK-242 treatment on LPS-induced splenocytes proliferation. hSOD1^G93A^ transgenic mice were treated with TAK-242, a selective TLR4 inhibitor, or vehicle. Survival, body weight, and motor behavior were monitored. To evaluate in vivo immunologic modifications associated with TAK-242 treatment, we measured serum IL-1β in the plasma, as well as IL-1β and TNF-α mRNAs in the spinal cord in wild-type mice and in TAK-242-treated and vehicle-treated early symptomatic hSOD1^G93A^ mice. Immunohistochemical analysis of motor neurons, astrocytes, and microglial reactivity in the spinal cords were performed on symptomatic (100 days old) TAK-242-treated and vehicle-treated hSOD1^G93A^ mice. In vitro, splenocytes taken from 100 days old hSOD1^G93A^ mice showed significantly increased proliferation when exposed to LPS (*p* = 0.0002), a phenomenon that was reduced by TAK-242 (*p* = 0.0179). TAK-242 treatment did not attenuate body weight loss or significantly affect survival. However, TAK-242-treated hSOD1^G93A^ mice showed temporary clinical delay in disease progression evident in the ladder test and hindlimb reflex measurements. Plasma IL-1β levels were significantly reduced in TAK-242-treated compared to vehicle-treated hSOD1^G93A^ mice (*p* = 0.0023). TAK-242 treatment reduced spinal cord astrogliosis and microglial activation and significantly attenuated spinal cord motor neuron loss at early disease stage (*p* = 0.0259). Compared to wild-type animals, both IL-1β and TNF-α mRNAs were significantly upregulated in the spinal cords of hSOD1^G93A^ mice. Spinal cord analysis in TAK-242-treated hSOD1^G93A^ mice revealed significant attenuation of TNF-α mRNA (*p* = 0.0431), but no change in IL-1β mRNA. TLR4 inhibition delayed disease progression, attenuated spinal cord astroglial and microglial reaction, and reduced spinal motor neuron loss in the ALS hSOD1^G93A^ mouse model. However, this effect did not result in increased survival. To our knowledge, this is the first report on TAK-242 treatment in a neurodegenerative disease model. Further studies are warranted to assess TLR4 as a therapeutic target in ALS.

## 1. Introduction

Amyotrophic lateral sclerosis (ALS) is a neurodegenerative, incurable, lethal disease, characterized by irreversible degeneration of upper and lower motor neurons in the brain and spinal cord [1,2]. Progressive loss of motor neurons leads to muscle atrophy, paralysis, and death from respiratory muscle failure [1]. ALS occurs worldwide, with an estimated annual incidence of 2–3 per 100,000 [3]. Between 5% and 10% cases of ALS are familial, of which mutations in the copper/zinc ion-binding superoxide dismutase-1 (SOD1) gene account for 20% [3]. SOD1 mutations also account for 2–3% of apparently sporadic cases of the disease [4]. The most commonly used rodent model of ALS is a transgenic mouse harboring the G93A SOD1 mutation, in which glycine is replaced by alanine at position 93 of the human SOD1 gene (hSOD1^G93A^ mouse). 

Neuroinflammation contributes to disease progression in many neurodegenerative diseases, including ALS [5,6]. Toll-like receptors (TLRs) have an important part in the formation of chronic inflammation in these illnesses [5,7].

TLRs, which constitute part of the innate immune system, are transmembrane receptors expressed by different immune cells [8]. These receptors recognize structures essential for the life cycle of pathogens called pathogen-associated molecular patterns (PAMPs) [7]. In addition, TLRs recognize endogenous molecules termed danger-associated molecular patterns (DAMPs) [5,9]. To date, 13 mammalian TLRs are recognized, of which TLR1–TLR10 are conserved between human and mice, whereas TLR11–TLR13 are not found in humans [7,8]. TLRs 1, 2, 4, 5, 6, and 11 are extracellular receptors localized on the cell surface, while TLRs 3, 7, 8, and 9 are intracellular, mainly in endosomal and lysosomal compartments as well as in the endoplasmic reticulum [7]. TLRs recognize different ligands by their N-terminal outer membranous part, the leucine-rich repeat domain [8]. Adaptor molecules are recruited to the intracellular C-terminus section of the receptor, the Toll-interleukin1 receptor (TIR) domain, for further downstream signal transduction [8]. Depending on the particular TLR, receptor activation can induce nuclear factor κ-B (NF-κB), mitogen-activated kinases, and/or interferon-regulatory factor pathways, which regulate the expression of different genes involved in the inflammatory response [9].

In the central nervous system (CNS), Toll-like receptor-4 (TLR4) is expressed on microglia, astrocytes, and neurons [7]. The PAMP recognized by TLR4 is lipopolysaccharide (LPS), the main component of cell wall on Gram-negative bacteria [7,8]. A wide array of endogenous DAMPs may trigger TLR4 activation, including high-mobility group box-1 (HMGB1), amyloid-beta, fibronectin, and others [7]. Most DAMPs are sequestered as a consequence of the immune response. However, during CNS disease, self-antigens and/or danger signals may be released as a result of cell death, necrosis, or tissue remodeling [9]. Damaged neurons may activate microglia through the release of injury signals. The interaction between microglia and neurons may lead to self-amplification of neuronal injury and microglial activation, resulting in neurodegenerative disease [7]. It has been shown that TLR2 and TLR4 expressed on microglia are associated with neuroinflammation, clearance of aggregate proteins involved in neurodegenerative disorders and the progression of the neurodegenerative process in Alzheimer’s disease and in Parkinson’s disease [5,10,11]. Microglia and astrocytes activation also affect the progression of ALS [6,12]. 

A few recent studies implicated TLR4 activation as an important factor in the pathogenesis of ALS. These studies used either TLR4 knockout mice [2] or antagonists [13,14]. Compared to wild-type (WT) mice, TLR4 and HMGB1 were increased during disease progression in the spinal cords of hSOD1^G93A^ mice. These were measured both by mRNA expression and protein expression in different disease stages [2]. In the same study it was also shown that TLR4 and HMGB1 were localized to activated microglia and astrocytes, and that hSOD1^G93A^ mice lacking TLR4 had small transient improvement in hind-limb grip strength and significantly extended survival when compared to TLR4-sufficient hSOD1^G93A^ mice [2]. TLR4 antagonists have been shown to prevent wild-type motor neuron death in co-cultures with glia from hSOD1^G93A^ mice [14], and mice treated with the cyanobacteria-derived TLR4 antagonist VB3323 achieved better motor performance compared to vehicle-treated mice [13].

TAK-242 (Resatorvid) is an exogenous synthetic selective inhibitor of TLR4, which binds to the TIR domain of the receptor and disrupts the interaction of TLR4 with its adaptor molecules, thereby inhibiting the downstream signaling events [15]. TAK-242 was shown to protect against acute cerebral ischemic-reperfusion injury [16] and to reduce intracerebral hemorrhage-induced brain injury in mice [17].

This study was aimed to test the effects of prolonged treatment with the selective TLR4 inhibitor, TAK-242, on disease progression and survival in the hSOD1^G93A^ mouse model of ALS.

## 2. Results

### 2.1. TAK-242 Attenuated Splenocytes Proliferation in hSOD1^G93A^ Mice

To test whether TAK-242, a specific TLR4 inhibitor, could be biologically active in hSOD1^G93A^ mice, we performed an in vitro experiment, in which we measured LPS-induced reaction of splenocytes obtained from early symptomatic, 100 days old hSOD1^G93A^ mice (see materials and methods).

**Figure 1:**
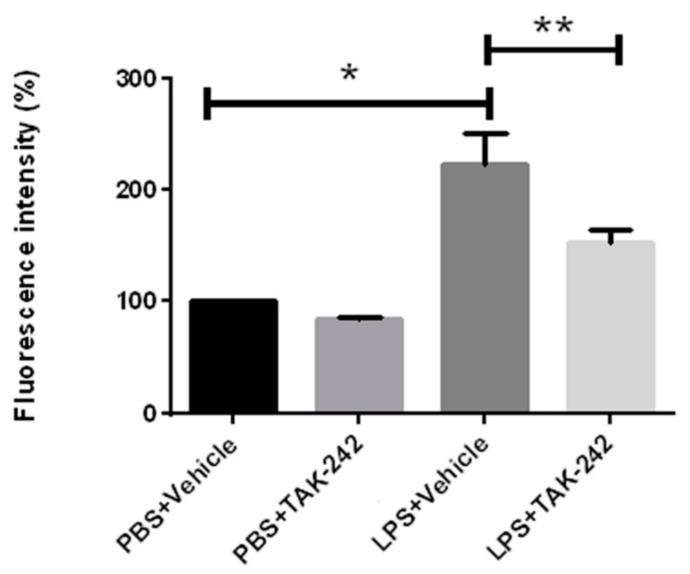
LPS-induced splenocyte proliferation in vitro was significantly reduced by TAK-242 treatment. Splenocytes obtained from 100 days old hSOD^G93A^ mice were treated with TAK-242 or vehicle for three days. Proliferation was measured from the Alamar blue assay and quantified by fluorescence intensity. PBS: phosphate-buffered saline; LPS: lipopolysaccharide. Data are expressed as mean ± SEM, * *p* < 0.01, ** *p* < 0.05.

TAK-242 treatment did not significantly attenuate proliferation of splenocytes exposed to phosphate-buffered saline (PBS). Splenocytes exposed to LPS showed significantly increased proliferation (*p* = 0.0002). The cells’ proliferative response after LPS exposure was significantly reduced by TAK-242 treatment (*p* = 0.0179; Figure 1). 

### 2.2. TAK-242 Treatment Transiently Delayed Clinical Disease Progression in hSOD1^G93A^ Mice

**Figure 2:**
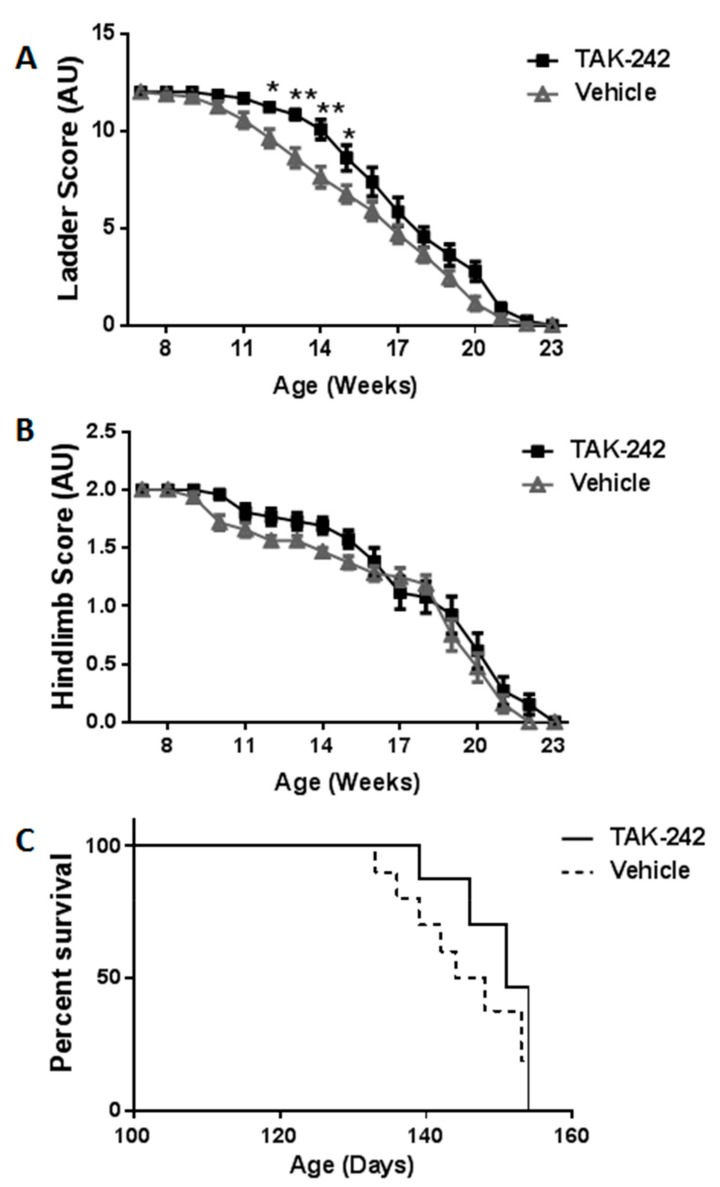
Behavioral motor performance analysis showed delay in disease progression in TAK-242- treated hSOD^G93A^ mice. (**A**) The ladder test. A score of 12 represents completely healthy mice and 0 correlates with disease end stage. Ladder testing showed statistically significant differences between hSOD^G93A^ mice treated with TAK-242 or vehicle. Data are expressed as mean ± SEM, * *p* < 0.05, ** *p* < 0.01; (**B**) A similar trend in disease progression was found in hind limb reflex score testing. Data are expressed as mean ± SEM; (**C**) Survival analysis by Kaplan–Meier curves of TAK-242 and vehicle-treated hSOD^G93A^ mice. All wild-type littermates survived and kept having a normal maximal score in all behavioral tests during the whole experiment period (not shown in the graphs).

Next, we tested the therapeutic effects of in vivo administration of TAK-242 on disease progression and survival of hSOD1^G93A^ mice, a well-established ALS model [18]. Mice were treated three times a week from the age of 7 weeks with TAK-242 3 mg/kg (13 mice) or vehicle (16 mice), as described in the methods section. Body weight and motor behavior were followed. No significant differences were detected in body weight or in gait score between hSOD1^G93A^ mice treated with TAK-242 or vehicle. However, motor behavioral testing showed attenuation of disease progression in TAK-242-treated hSOD1^G93A^ mice as compared to vehicle treated hSOD1^G93A^ mice. Ladder testing showed improved results in treated mice through weeks 11–20 of life, with statistically significant difference between the two groups in weeks 12–15 of life (Figure 2A). A similar, although not statistically significant, temporary delay in disease progression was detected by hindlimb reflex testing. TAK-242-treated hSOD1^G93A^ mice had delayed deterioration of this reflex in weeks 12–16 of life as compared to vehicle-treated hSOD1^G93A^ mice (Figure 2B). As shown in Figure 2C, although a non-significant trend for delay in the first death events was found in TAK-242-treated hSOD1^G93A^ mice, survival was not significantly extended in these animals as compared to vehicle-treated mice. All WT littermates survived and kept having a normal maximal score in all behavioral tests during the whole experiment period.

### 2.3. TAK-242 Treatment Decreased Serum IL1-β Levels in hSOD1^G93A^ Mice

**Figure 3:**
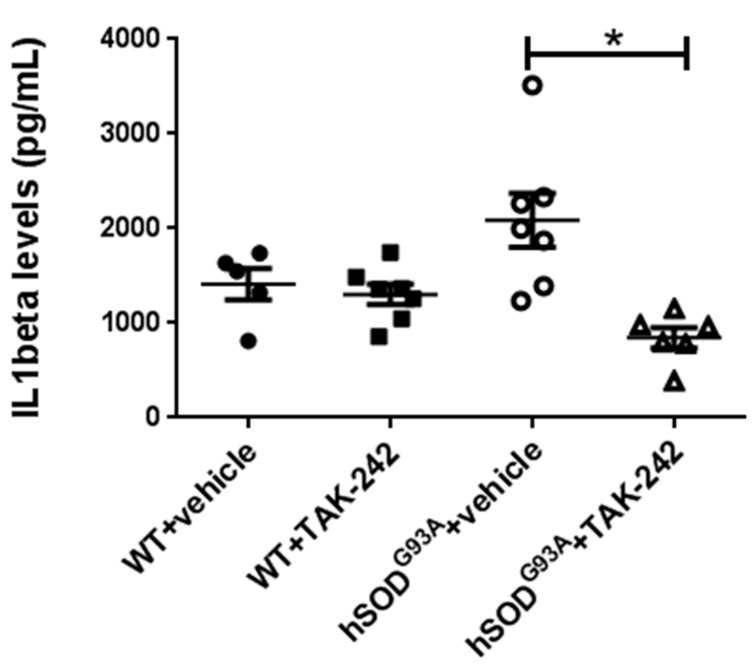
Prolonged TAK-242 treatment attenuates serum IL1-β levels of early symptomatic hSOD^G93A^ mice. IL1-β levels were measured by ELISA testing in sera obtained at day 100 of life, after 50 days of treatment with TAK-242 or vehicle. Analysis showed an increase in IL1-β levels in vehicle-treated hSOD^G93A^ mice compared to WT mice, which was significantly attenuated by TAK-242 treatment. WT: wild type. * *p* < 0.01.

In order to evaluate the effect of prolonged in vivo TAK-242 treatment on disease and immune activities we examined these effects in 100-days old (early symptomatic disease stage) hSOD1^G93A^ mice treated with TAK-242 (6 mice) or vehicle (7 mice). WT littermates were used as controls. Mice were treated from the age of 7 weeks, as described in the methods section. IL1-β ELISA testing of sera obtained after 50 days of treatment, at the age of 100 days, showed an increase in IL1-β levels in vehicle-treated hSOD1^G93A^ mice compared to WT mice. IL1-β was significantly decreased in the sera of TAK-242 treated hSOD1^G93A^ mice as compared to vehicle-treated hSOD1^G93A^ mice (*p* = 0.0023; Figure 3). This result demonstrates that prolonged in vivo treatment with TAK-242 has an immunomodulatory effect in hSOD1^G93A^ mice.

### 2.4. TAK-242 Treatment Attenuated Motor Neuron Loss in the Spinal Cords of hSOD1^G93A^ Mice

Motor neuron loss was assessed by staining of spinal cord slices of 100-days old hSOD1^G93A^ mice treated with TAK-242 or vehicle since the age of 7 weeks. Motor neuron counts were done at the age of 100 days on H&E stained sections (9 sections from each animal; 3 animals in each group), and verified by staining for the motor neuron marker Choline Acetyltransferase (ChAT), as described in the methods section.

**Figure 4:**
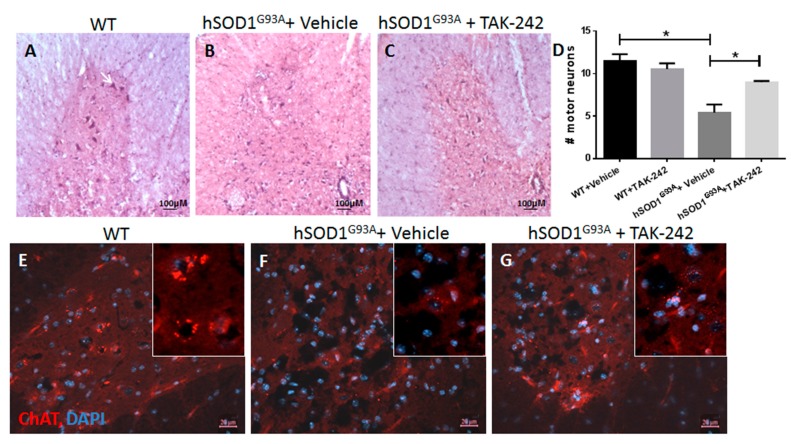
Preservation of spinal motor neurons in TAK-242-treated hSOD^G93A^ mice. Lumbar spinal cords sections (9 sections from each animal; 3 animals in each group) from early symptomatic hSOD^G93A^ mice (100 days old) were stained by H&E staining (**A**–**C**); Immunohistochemistry staining for the motor neuron marker ChAT is shown in (**E**–**G**); Motor neuron number was significantly reduced in hSOD^G93A^ mice treated with vehicle (**B**,**F**) compared to WT mice (**A**,**E**); Motor neuron loss was significantly reduced in hSOD^G93A^ mice treated with TAK-242 (**C**,**G**); Motor neurons quantification is shown in (**D**) and represents average number of motor neurons per one ventral horn in each group. An example of a motor neuron is marked by the arrow in A. WT: wild type; H&E: hematoxylin and eosin; ChAT: choline acetyltransferase; DAPI: 4,6-diamidino-2-phenylindole. Scale bars are 100 µm for (**A**–**C**) and 20 µm for (**E**–**G**). Data are expressed as mean ± SEM, * *p* < 0.05.

Spinal cord motor neuron number was significantly reduced in hSOD1^G93A^ mice as compared to WT healthy littermates (*p* = 0.0214). TAK-242 treatment significantly attenuated motor neurons loss in hSOD1^G93A^ mice (*p* = 0.0259). These results were detected by H&E motor neuron staining (Figure 4A–D) and supported by ChAT immunohistochemical staining (Figure 4E–G). These results imply motor neuron preservation by TAK-242 treatment, in parallel to the temporary delay in motor deterioration in the early symptomatic stage detected by behavioral tests as described above (Figure 2A,B).

### 2.5. TAK-242 Treatment Attenuated Spinal Cord Astrogliosis and Microglia Activation in hSOD1^G93A^ Mice

**Figure 5:**
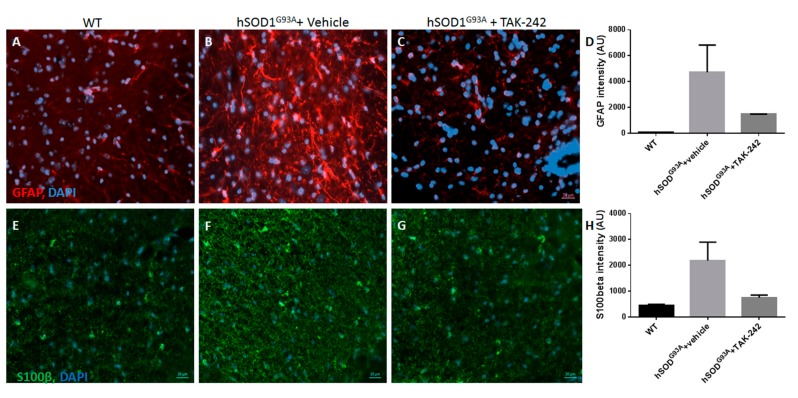
Prolonged TAK-242 treatment attenuates astroglial activation in hSOD^G93A^ mice spinal cords. Immunohistochemistry staining for the astrocyte markers GFAP (red; **A**–**C**) and S100β (green; **E**–**G**) at day 100 of life showed increased spinal astrogliosis in vehicle-treated hSOD^G93A^ mice (**B**,**F**) compared to WT littermates (**A**,**E**), that was prominently attenuated in TAK-242 treated hSOD^G93A^ mice (**C** (*p* = 0.0462); **G** (*p* = 0.0481)). Quantification of staining intensity is shown graphically in panels **D** (GFAP) and **H** (S100β). WT: wild type; GFAP: glial fibrillary acidic protein; DAPI: 4,6-diamidino-2-phenylindole. Scale bars for all panels = 20 µm.

**Figure 6:**
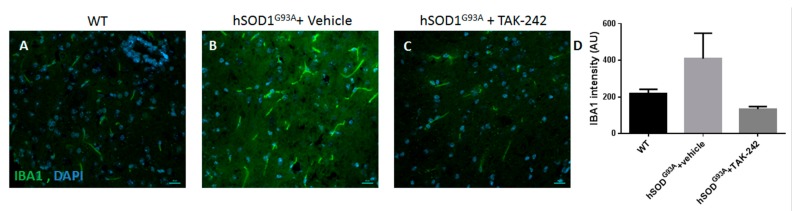
Prolonged TAK-242 treatment attenuates microglial activation in hSOD^G93A^ mice spinal cords. Immunohistochemistry staining for the activated microglia marker IBA1 at day 100 of life showed increased spinal microglial activation in vehicle-treated hSOD^G93A^ mice (**B**) as compared to WT littermates (**A**). Microglial activation was prominently attenuated by prolonged TAK-242 treatment of hSOD^G93A^ mice (**C**; *p* = 0.0544). Quantification of staining intensity is shown graphically in panel (**D**). WT: wild type; IBA1: ionized calcium-binding adapter molecule-1; DAPI: 4,6-diamidino-2-phenylindole. Scale bars for all panels = 20 µm.

Histological analysis of the spinal cord sections of 100-days old hSOD1^G93A^ mice showed increased astroglial and microglial reactivity (Figure 5 and Figure 6), as described previously [19]. TAK-242 treatment prominently attenuated the pathological astrogliosis and microgliosis (Figure 5 and Figure 6).

Immunohistochemical staining for glial fibrillary acidic protein (GFAP) and S100β were used as astrocytic markers, and ionized calcium-binding adapter molecule 1 (IBA1) as a microglial marker. We found marked increase in astrogliosis and microglial activation in vehicle-treated hSOD1^G93A^ mice as compared to their age-matched WT littermates (Figure 5 and Figure 6). Prolonged in vivo treatment with TAK-242 of hSOD1^G93A^ mice, from the age of 7 weeks until the age of 100 days, significantly attenuated astrogliosis, as shown in Figure 5, and microglial reaction, as shown in Figure 6.

### 2.6. TAK-242 Treatment Attenuated TNF-α but Not IL1-β Expression in the Spinal Cords of hSOD1^G93A^ Mice

**Figure 7:**
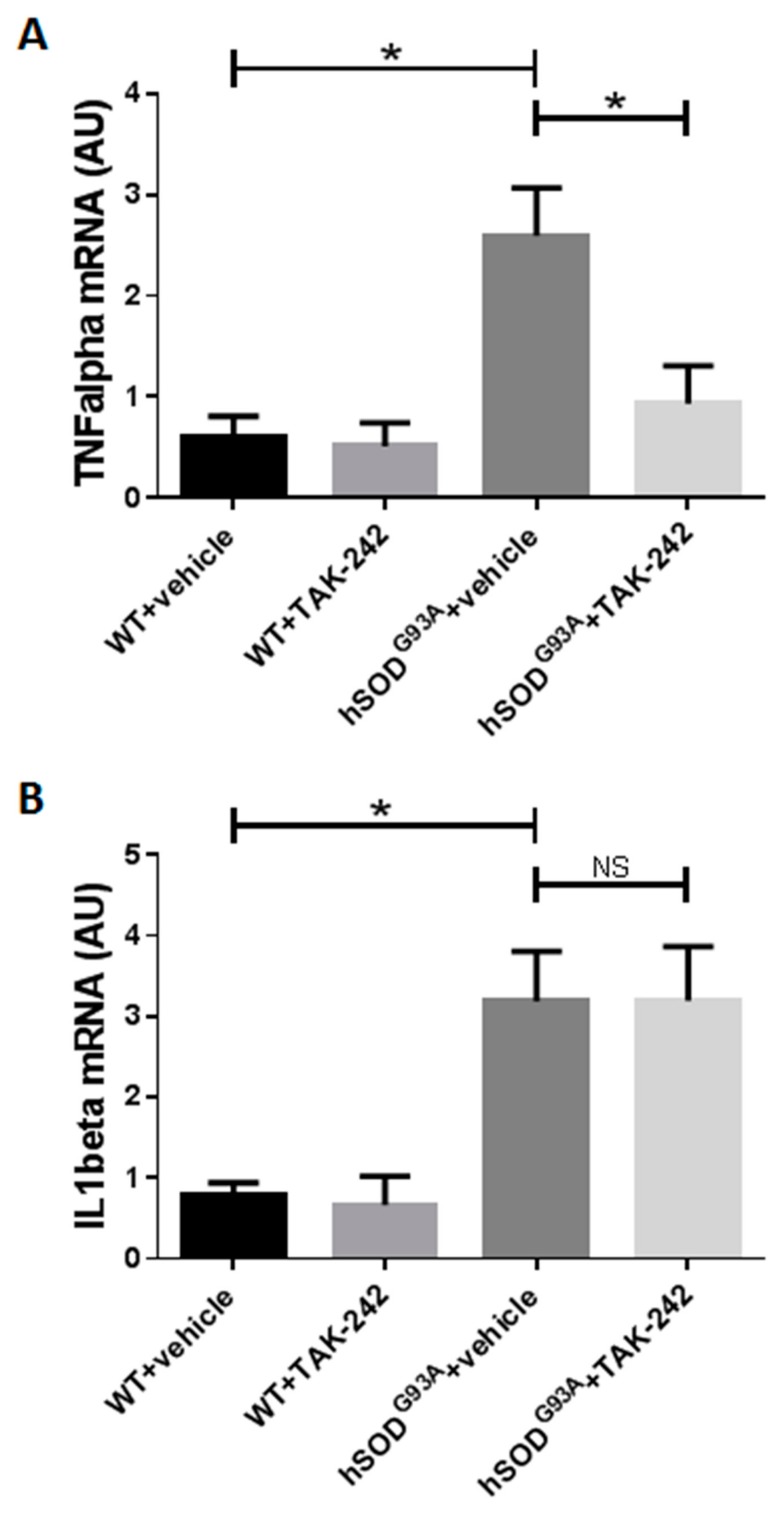
Prolonged TAK-242 treatment attenuated TNF-α expression but not IL1-β expression in the spinal cords of early symptomatic hSOD^G93A^ mice. TNF-α (**A**) and IL1-β (**B**) mRNA levels in the spinal cords of TAK-242-treated and vehicle-treated 100-days old WT and early symptomatic hSOD^G93A^ mice were quantified by real-time PCR as described in the methods section. WT: wild type; NS: non-significant. Data are expressed as mean ± SEM, * *p* < 0.05.

In order to evaluate the effect of prolonged in vivo TAK-242 treatment on spinal cord inflammatory cytokines, we examined this effect in 100-days old (early symptomatic disease stage) hSOD1^G93A^ mice treated with TAK-242 or vehicle, as well as in their WT littermates (3 animals in each group). Mice were treated from the age of 7 weeks and quantitative PCR analysis was performed as described in the methods section. Compared to WT mice, real-time quantitative PCR (RT-PCR) demonstrated significant mRNA upregulation in the spinal cords of vehicle-treated hSOD1^G93A^ mice for both TNF-α (*p* = 0.0167) and IL1-β (*p* = 0.0174). When comparing TAK-242-treated hSOD1^G93A^ mice and vehicle-treated hSOD1^G93A^ mice, TNF-α mRNA level in the spinal cords was significantly decreased in TAK-242 treated animals (*p* = 0.0431), and no difference was found in IL1-β mRNA level between the two groups (Figure 7). These results suggest that the beneficial effects in the spinal cord of TAK-242 treatment observed in our study involved attenuation of TNF-α-mediated motor neuron injury with no change in the local spinal cord IL1-β levels.

## 3. Discussion

Accumulating evidence implies that inflammatory response may play a significant role in motor neuron injury pathogenesis in ALS [6,12,20], and the innate immune system has been found to contribute to this process [6,20]. The TLR system is an important component of innate immunity. Toll-like receptor-4 (TLR4) is a central pro-inflammatory TLR found in numerous types of cells, including CNS cells. Our present study examined the effects of prolonged in vivo TLR4 inhibition by TAK-242 treatment in a mouse model of ALS (hSOD1^G93A^). TAK-242 treatment resulted in moderate attenuation of motor function decline, preservation of motor neuron survival, and a decrease in parenchymal gliosis and microglial activation. However, there was no prolongation of survival.

Many studies have characterized the activation of microglia and astrocytes in post-mortem tissue of patients with ALS and in spinal cords of transgenic mice that express the mutant form of human SOD1 [20]. Upregulated expression of CD11b, IBA1, and CD68 markers for microglia, and of GFAP and Aldehyde dehydrogenase 1 family member L1 markers for astrocytes, is reported consistently [20], implying that activation of astrocytes and microglia may have a role in ALS pathogenesis. In addition, the morphological appearance of these cells is changed from a surveying state, characterized by a small cell body and thin processes, to an activated state, in which the cell body is enlarged and the processes are thickened [20]. A recent study showed that glial activation colocalizes with structural abnormalities in ALS patients [21].

Activated microglia are found at sites of neurodegeneration in brain and spinal cord tissues from ALS patients [22]. The importance of microglial activation in disease pathogenesis was demonstrated in a study showing that microglial NF-κB activation is required for motor neuron death induction in hSOD1^G93A^ mouse model of ALS [23]. In primary cultures of motor neurons and microglia, extracellular mutant SOD1 was found to induce microglial morphological and functional activation, causing motor neuron injury [24]. It has also been shown that the extent of microglial activation in the corticospinal tract of ALS patients correlates with disease progression and is linked to upper motor neuron deficits [25].

There is also evidence to support the role of astrocytes in inducing neurotoxicity in ALS [26]. Astrocytes from post-mortem CNS of ALS patients were neurotoxic to co-cultured motor neurons [27,28]. Additionally, it was reported that astrocytes generated from sporadic and familial (C9ORF72 and SOD1) ALS patients induced pluripotent stem cells were also toxic to co-cultured motor neurons [29].

In an attempt to find therapeutic targets in the innate immune system that would allow disease modification, the role of TLRs in ALS has been studied during recent years. Upregulated expression of TLR2 and TLR4 as well as increased expression of HMGB1, a major endogenous ligand of TLR4, were demonstrated in spinal cord specimens of patients with sporadic ALS [30]. TLR4 and HMGB1 were increased during disease progression in the spinal cords of hSOD1^G93A^ mice, with TLR4 and HMGB1 localized to activated microglia and astrocytes [2]. It was also found that hSOD1^G93A^ mice lacking TLR4 had small transient improvement in hind-limb grip strength and significantly extended survival when compared to TLR4-sufficient hSOD1^G93A^ mice [2]. TLR4 antagonists prevented motor neuron death in co-cultures with glia from hSOD1^G93A^ mice [14], and mice treated with the cyanobacteria-derived TLR4 antagonist VB3323 achieved better motor performance compared to vehicle-treated mice [13].

In this study we further investigated the importance of TLR4 in the pathogenesis of ALS, using a selective TLR4 antagonist, TAK-242, in the hSOD1^G93A^ mouse model of this disease. TAK-242 has been previously tested in a clinical trial for the treatment of sepsis [31], and in animal models for different conditions, such as acute cerebral ischemia/reperfusion injury [16] and hemorrhage-induced brain injury [17]. To our knowledge, this is the first time TAK-242 is being tested for the treatment of a neurodegenerative disease. Another unique feature of this trial is the prolonged in vivo treatment with TAK-242. 

We found temporary clinical delay in disease progression in hSOD1^G93A^ mice treated with TAK-242. As in previous studies [2,13], this delay did not affect weight decline but was evident in motor behavioral tests. In parallel to the temporary delay in motor decline, histological examination of spinal cord sections from hSOD1^G93A^ mice in the early symptomatic stage showed statistically significant attenuation of motor neuron loss in TAK-242-treated mice, as compared to vehicle-treated littermates.

We also found that TAK-242 treatment attenuated the increased activation of spinal astrocytes and microglia in hSOD1^G93A^ mice, in parallel with the temporal clinical delay in disease progression. These findings further support previously published data which implied a role for microglia and astrocyte activation in the pathogenesis of disease progression in ALS.

When combining our findings with previous data that suggested a role for astrocytic and microglial activation in ALS pathogenesis, it is possible that clinical and histological beneficial effects found in parallel to attenuation of the increased glial activation in the spinal cord of TAK-242-treated hSOD1^G93A^ mice, suggests a beneficial neuroprotective effect of this treatment, possibly mediated by the attenuation of spinal cord glial activation. Nevertheless, the particular mechanism by which TAK-242 is neuroprotective in this animal model is not clear. The changes demonstrated in our study in serum IL1-β and in TNF-α and IL1-β expression in the spinal cord may partially elucidate a certain aspect of this mechanism. The significant attenuation of increased serum IL1-β levels in TAK-242-treated hSOD1^G93A^ mice shows that prolonged TAK-242 treatment in vivo has an immunomodulatory effect in the peripheral blood. However, when examining the local cytokine expression in the spinal cord, while finding a significant increase in both IL1-β and TNF-α in vehicle-treated hSOD1^G93A^ mice compared to their WT littermates, which was also shown previously [32,33], we found a significant attenuation only in TNF-α expression and no effect on IL1-β expression in the spinal cord of TAK-242-treated hSOD1^G93A^ mice compared to vehicle-treated hSOD1^G93A^ mice. These findings suggest that TNF-α is a local pro-inflammatory effector in the spinal cord attenuated by TAK-242 treatment, while IL1-β modulation by TAK-242 treatment in the peripheral blood may induce its neuroprotective effects through an indirect mechanism, with no significant direct influence on IL1-β expression in the spinal cord. This is in line with a previous study in which IL1-β gene knockout was used to demonstrate that IL1-β does not directly contribute to motor neuron degeneration, but may modulate the innate immune system in a mutant SOD1 model of ALS [34]. Butovski et al. have shown that recruitment of inflammatory monocytes from the peripheral blood to the spinal cord plays an important role in disease progression in mutant SOD1 mice, and that modulation of these cells attenuated neuronal loss and extended survival in this animal model [35]. Therefore, it is possible that the beneficial effect of TAK-242 treatment observed in our study was mediated, at least partially, by modulation of peripheral inflammatory cells by the treatment, an aspect to be addressed in an additional study in the future. In our study, we evaluated only IL1-β and TNF-α as inflammatory markers to assess the immunomodulatory effects of TAK-242 treatment in vivo. Since TLR4 antagonism by TAK-242 may affect other pro-inflammatory and anti-inflammatory cytokines, further studies should include measurements of other cytokines in order to better understand TAK-242 effects in the hSOD1^G93A^ mouse model. Additional aspects of the TAK-242 mechanism of action in this model warrant further investigation as well. One possibility, for example, would be to perform in vitro examination of TAK-242 in microglial cultures from hSOD1^G93A^ mice.

It is notable, however, that we found only a moderate and temporary clinical delay in disease progression with no impact on survival in TAK-242-treated animals. A possible explanation for obtaining only a moderate, temporary effect by the treatment may be related to the dosing of the drug. Since this was the first time TAK-242 was used in a neurodegenerative disease model and the previous data regarding TLR4 antagonism in ALS are limited, we chose the dosage and administration schedule based on the limited data in the literature. TAK-242 dosage (3 mg/kg) was based on previous studies in mouse models for other conditions that used the same dosage [16,17]. Due to limited data regarding long-term TAK-242 administration schedule in chronic disease models, we used a three times per week administration schedule based on a previous study that tested another TLR4 antagonist in an ALS mouse model [13]. TLR4 knockout was found to significantly extend survival in hSOD1^G93A^ mice [2]. Since an exogenous chemical manipulation of the receptor is not as robust as receptor knockout, it is possible that different TAK-242 dosing is needed to achieve a more significant effect. This may be addressed in an additional study in the future. In our study, we did not investigate the peripheral effects of TAK-242 treatment on innervation or neuromuscular junction (NMJ). Since peripheral denervation occurs before motor neuron death and symptoms onset in ALS [36], this process may have begun prior to or around the start of TAK-242 therapy, thereby limiting the beneficial impact of the treatment. Therefore, future studies assessing TAK-242 treatment in ALS should address treatment effects on peripheral innervation and NMJ integrity. Another possible explanation for achieving only a moderate beneficial effect by TLR4 inhibition claims that neuroinflammation is only a part of the disease process in ALS, and that the neurodegenerative process is relentlessly progressing even without the added damage induced by the accompanying inflammatory response.

An important aspect of this study is the prolonged in vivo TAK-242 treatment. Previous studies tested TAK-242 for acute conditions, with short-lasting use of this drug up to several days. To our knowledge, this is the first time TAK-242 is used in a neurodegenerative disease model, therefore requiring long-lasting administration of the drug over a period of about three months (median 97 days). We have not observed any major side effects of the drug during this study. However, if TAK-242 is to be considered as a potential chronic treatment for ALS or other neurodegenerative disorders, then its adverse effects in long-term use should be further evaluated in additional studies in the future.

## 4. Materials and Methods

### 4.1. Transgenic hSOD1^G93A^ Mice

Mice overexpressing the human mutant superoxide dismutase 1 (hSOD1^G93A^), a well-established ALS model [18], were purchased from Jackson Laboratories (Bar Harbor, ME, USA). The animals were kept in standard conditions of constant temperature (22 ± 1 °C), humidity (relative, 40%) and a 12-h light/dark cycle, and were given free access to food and water. Male mice hemizygous for the mutant hSOD1^G93A^ transgene, which results in a dominant disease phenotype, were bred with control C57Bl/6J females. This way each litter generated hemizygous hSOD1^G93A^ transgenic mice and littermate controls. D-Tail DNA extraction kit (Syntezza Bioscience, Jerusalem, Israel) was used to extract genomic DNA from tail biopsies. Offspring mice were genotyped by PCR analysis using primers for IL2 and human SOD1 as shown in Table 1.

### 4.2. Treatment Protocol

Hemizygous hSOD1^G93A^ transgenic mice were treated starting at the age of 7 weeks, intraperitoneally, with vehicle (saline) or TAK-242 (ApexBio, Houston, TX, USA; concentration: 0.3 mg/mL; dosage: 3 mg/kg), three times per week. This protocol was based on previous in vivo experiments with TAK-242 and with other TLR4 antagonists [13,16,17]. The experiments were performed in accordance with local and international regulations, and were approved by the Tel Aviv University Ethical Committee (Identification code: M-15-094; Date of approval: 24 November 2015).

### 4.3. Behavioral Motor Evaluations

Follow-up and treatment continued until end stage definition (13 TAK-242 treated hSOD1^G93A^ mice (5 males, 8 females); 16 vehicle-treated hSOD1^G93A^ mice (8 males, 8 females)). Body weight was measured weekly starting from week 7 and twice a week from moderate disease stage until end stage.

To determine death in a reliable and ethical fashion, starting from age 14 weeks the mice were tested daily to determine if they have reached end stage, which was defined by the inability of the animal to right itself within 30 s of being placed on its side. Behavioral assessments were performed in parallel by the same observer. Behavioral tests included the ladder test, gait scoring, and hindlimb reflex measurements.

#### 4.3.1. Ladder Test

To evaluate the animal’s overall neurological state, we performed a neurological scoring based on the ladder test, as described before [37]. The ladder is placed at a 45° angle. After a brief training period, when placed on the ladder, healthy mice climb up the ladder quickly and efficiently. The animals’ ability to climb is hindered with disease progression, initially with leg tremors, followed by gradual deterioration until the mice are unable to climb up the ladder. The animals were scored based on their performance on the ladder test. A score of 12 represents completely healthy mice and 0 correlates with end stage disease.

From the age of 7 weeks, the mice were evaluated weekly for their ability to climb the ladder and were scored weekly. The score was determined using the guidelines described in Table 2.

#### 4.3.2. Neurological Gait Assessment

Gait evaluation was based on a well accepted 5-point scoring system [38]. Gait was scored as follows: healthy with no symptoms of paralysis (score—5); mild signs of destabilized gait and paralysis of the hind limbs (score—4); obvious paralysis with abnormal gait (score—3); full paralysis of the hind limbs, animals only crawling on their forelimbs (score—2); full paralysis of the hind limbs, animals predominantly lying on their side and/or unable to straighten up after turning them on the back, or lost more than 20% of their initial weight (score—1). When reaching a score of 2, animals were given macerated food daily for easier access of food and hydration. Gait was evaluated weekly and scored starting from the age of 7 weeks until end stage or death.

#### 4.3.3. Hindlimb Reflex Measurement

Healthy mice reflexively extend their legs backwards when lifted by their tail, in order to improve their balance. With disease progression, hSOD1^G93A^ mice lose the ability to extend their legs and this reflexive extension is gradually lost. From the age of 7 weeks, the mice were evaluated weekly for their hind leg reflexes. Hindlimb extension reflexes were evaluated as described previously [39]. Mice were suspended by the tail, and the degree of motor deficits in the hind limbs was scored from 0 to 2 as follows: a normal extension reflex in both hind limbs—2; imbalanced extension in the hind limbs—1.5; extension reflex in only one hind limb—1.0; the absence of any hind limbs extension—0.5; and total paralysis was scored 0.

### 4.4. Plasma IL1-β Measurement

At the age of 100 days, 6 TAK-242-treated and 7 vehicle-treated hSOD1^G93A^ mice were sacrificed and their blood was collected. Seven TAK-242-treated and 5 vehicle-treated WT littermates were used as controls. Plasma IL1-β concentration was measured by sandwich ELISA procedure, according to the manufacturer’s instructions (Mini ABTS EDK-murine IL1-beta; PeproTech, Rocky Hill, NJ, USA). The absorbance at 405 nm with wavelength correction at 650 nm was measured by a microplate reader (PowerWaveX; BioTek Instruments, Winooski, VT, USA).

### 4.5. Histological Analysis

hSOD1^G93A^ mice treated with TAK-242 or vehicle (3 animals in each group) were anesthetized and sacrificed at the age of 100 days, at an early symptomatic disease stage. WT littermates were used as controls. Mice were transcardially perfused with PFA 4%. The rostral part of the spinal cord was frozen for further analysis, and the caudal part was used for histological motor neuron analysis and immunohistochemistry. Lumbar spinal cords were dissected, embedded in optimal cutting temperature (OCT, Tissue-Tek; Scigen Scientific, Gardena, CA, USA), sectioned (10 μm) using a cryostat, and stained with Hematoxylin and Eosin (H&E). To compare the number of motor neurons, we counted the large motor neurons (greater than 25 µm in diameter) in the ventral horn gray matter in 9 lumbar spinal cord sections from each animal. The results of motor neuron counting were expressed as the average of total motor neurons counted per one ventral horn in each group. For immunohistochemistry, slides were incubated with blocking solution (5% goat serum, 1% Bovine serum albumin, 0.05% Triton-X in phosphate-buffered saline (PBS)) for 1 h. Then, slides were incubated overnight at 4 °C with the following primary antibodies: rat anti-GFAP (1:500; Invitrogen, Carlsbad, CA, USA), goat anti-ChAT (1:500; Abcam, Cambridge, UK), mouse anti-S100β (1:10; Abcam), and goat anti-IBA1 (1:200; Abcam). Then, sections were incubated with the secondary antibody Alexa Fluor anti goat-488, anti-mouse-488, or anti rat-568 (1:200–1:1000; Invitrogen) for 1 h. The nuclei were counterstained with 4,6-diamidino-2-phenylindole (DAPI; 1:1000; Sigma-Aldrich, St. Louis, MO, USA). Microscopic pictures were taken by ApoTome microscope (ZEISS, Jena, Germany). ZEN pro 2012 software was used for staining intensity quantification.

### 4.6. IL1-β and TNF-α mRNA Measurements in the Spinal Cord

RNA extraction from the frozen spinal cords, cDNA synthesis and RT-PCR analysis was performed as previously described [40]. Assays were conducted according to the manufacturer’s specifications (Applied Biosystems, Foster city, CA, USA). IL1-β and TNF-α gene expression levels in the spinal cord were determined in TAK-242-treated and vehicle-treated hSOD1^G93A^ mice and in WT littermates (3 animals in each group), utilizing SyBr RT-PCR with the specific primers as shown in Table 3. Analysis and quantification were conducted using 7700 system software and compared to the expression of the housekeeping gene GAPDH.

### 4.7. Splenocytes Viability and Proliferation Assay

Four hSOD1^G93A^ mice were sacrificed at the age of 100 days by cervical dislocation. Following removal and mechanical dissociation of the spleens, splenocytes were washed twice with PBS and placed in RPMI 1640 medium supplemented with 50 µM 2-mercaptoethanol, 2 mM glutamine, antibiotic agents (100 U/mL penicillin G, 100 µg/mL streptomycin), and 10% heat-inactivated fetal calf serum (all from Biological Industries) at 37 °C. Splenocytes were then counted and plated at a concentration of 2 × 10^5^ cells/well in medium containing saline or TAK-242 (100 nM; ApexBio, Houston, TX, USA; concentration used was based on data published previously [41]) and were exposed to PBS or 20 µg/mL lipopolysaccharide (LPS; Difco, Detroit, MI, USA). Following incubation for 72 h at 37 °C in humidified air containing 5% CO_2_, Alamar blue 10% (AbD Serotec, Kidlington, UK) was added to the cells for an additional 3 h. Alamar blue assay is a sensitive quantitative assay assessing the conversion of the reagent by metabolically active cells into a fluorescent and colorimetric indicator. To assess cell numbers, we measured fluorescence intensity at an excitation wavelength at 560 nm and an emission wavelength at 590 nm using a fluorometer device (Synergy HT; BioTeck Instruments).

### 4.8. Statistical Analysis

Statistical analysis of the data sets was performed with GraphPad Prism software for Windows. Statistical significance was determined by two-way analysis of variance (ANOVA), one-way ANOVA, or t-test, as appropriate. Kaplan–Meier analysis was used to evaluate survival difference between the groups. All data are presented as mean ± standard error of the mean (SEM), error bars represent SEM, and minimal statistical significance level was set at *p* < 0.05.

## 5. Conclusions

We tested the role of TLR4 in the pathogenesis of ALS using the selective TLR4 inhibitor TAK-242 and hSOD1^G93A^ mice as the disease model. Our findings show that inhibition of TLR4 may be beneficial in ALS. Yet, the treatment effect in the current study was only moderate and temporary, and the findings need further examination to elucidate the possible role of TLR4 in ALS, as well as the optimal dosing of TAK-242. Further studies are required to elaborate our understanding of the role of neuroinflammation in ALS and of TLR4 as a potential therapeutic target in this lethal, incurable disease.

## Figures and Tables

**Table 1:** Primers used for PCR analysis of offspring mice.

Gene	Forward Primer	Reverse Primer
**hSOD1^G93A^**	CATCAGCCCTAATCCATCTGA	CGCGACTAACAATCAAAGTGA
**IL2**	CTAGGCCACAGAATTGAAAGATCT	GTAGGTGGAAATTCTAGCATCATCC

**Table 2:** Score and behavioral manifestation in the ladder test.

Score	Behavioral Manifestation
12	Pre-symptomatic, no visible difficulties climbing the ladder.
11	Early stage tremors, climbing speed not effected.
10	Increased tremors, difficulty in closing hind leg fingers around the stages of the ladder.
9	One of the hind legs begins to occasionally miss the stages of the ladder, climbing speed not affected.
8	Both of the hind legs begin to occasionally miss the stages of the ladder, climbing speed not affected.
7	One of the hind legs occasionally misses the stages of the ladder, climbing speed affected.
6	Both of the hind legs occasionally miss the stages of the ladder, climbing speed affected.
5	Climbing speed clearly affected, medium to slow speed, hind legs show symptoms.
4	Slow climbing speed, symptoms clearly visible in hind legs but not in front legs.
3	Slow climbing speed, symptoms visible in hind legs as well as in front legs.
2	The mouse climbs up the ladder very slowly and misses the stages of the ladder with all legs.
1	The mouse barely climbs up the ladder.
0	The mouse does not move once placed on the ladder.

**Table 3:** Primers used for RT-PCR analysis of the spinal cord.

Gene	Forward Primer	Reverse Primer
IL1-β	CCACCTCAATGGACAGAATATCA	CCCAAGGCCACAGGTATTT
TNF-α	GTCTCAGAATGAGGCTGGATAAG	CATTGCACCTCAGGGAAGAA
GAPDH	CGACAGTCAGCCGCATCTT	CCAATACGACCAAATCCGTTG

## Abbreviations

ALS	Amyotrophic lateral sclerosis
ChAT	Choline Acetyltransferase
CNS	Central nervous system
DAMP	Danger-associated molecular pattern
GFAP	Glial fibrillary acidic protein
HMGB1	High-mobility group box-1
IBA1	Ionized calcium-binding adapter molecule 1
LPS	Lipopolysaccharide
NF-κB	Nuclear factor kappa-B
NMJ	Neuromuscular junction
PAMP	Pathogen-associated molecular pattern
RT-PCR	Real-time polymerase chain reaction
SEM	Standard error of the mean
SOD1	Superoxidedismutase-1
TIR	Toll-interleukin 1 receptor
TLR	Toll-like receptor
WT	Wild-type
